# Effects of a Proprietary Kelp Blend Product on Enteric Methane Production and Tissue Residues in Cattle

**DOI:** 10.3390/ani14162411

**Published:** 2024-08-20

**Authors:** Alexander Altman, Eric Vanzant, Sydney Davis, David Harmon, Kyle R. McLeod

**Affiliations:** Department of Animal and Food Sciences, University of Kentucky, Lexington, KY 40546, USA; alexander.altman@uky.edu (A.A.); evanzant@uky.edu (E.V.); sydney.davis@uky.edu (S.D.); david.harmon@uky.edu (D.H.)

**Keywords:** cattle, methane mitigation, bromoform, residues, growth performance

## Abstract

**Simple Summary:**

The purpose of this study was threefold: (1) to quantify the suppressive effects of a proprietary bromoform-containing product on bovine enteric methane production after 11 days of supplementation, (2) to map the duration of these effects for up to 9 days post-supplementation, and (3) to investigate whether bromoform is incorporated into the edible tissues of cattle as a result of supplementation in the daily ration for 30 days. Bromoform was supplemented from 0 to 63 ppm of the diet. In this study, supplementing with bromoform product resulted in an approximate 98% reduction of enteric methane production, with reduced methane levels observed across a 9-day post-supplementation period as compared with animals not receiving bromoform. Feed consumption was not affected by bromoform product supplementation, and no bromoform residues were found in fat, organ, or muscle tissue samples collected upon animal harvest. Thus, it was concluded that low-level supplementation of this bromoform-containing product is beneficial in reducing bovine enteric methane production for short-term durations without accumulation of bromoform within organ and edible tissues and with no evidence of effects on animal growth or feed consumption.

**Abstract:**

Three experiments were performed investigating bovine enteric methane (CH_4_) production inhibition using a proprietary kelp blend product (PKBP) containing a halogenated methane analog (i.e., bromoform). Calves were fed a corn-silage basal diet top-dressed with the assigned treatment, with rations provided at 1.5 × NE_m_ in Experiments 1 and 2 (*n* = 12 and 6 steers, respectively) and ad libitum in Experiment 3 (*n* = 9 steers). In Experiment 1, we evaluated bromoform’s potency in decreasing CH_4_. Dry matter intake (DMI) was not affected by treatment (*p* ≥ 0.11; 0 vs. 52.5 ± 10.5 ppm bromoform), whereas bromoform supplementation decreased CH_4_ (*p* < 0.01). In Experiments 2 and 3, treatments were 0, 9.5 ± 1.5, or 20 ± 3 ppm bromoform. In Experiment 2, we examined CH_4_ recovery following bromoform removal from the ration. Bromoform treatments were fed on d1, but not the subsequent 8 d, to investigate residual effects. On d1, CH_4_ was below limits of detection for 20 ppm bromoform inclusion. Across days, a cubic response (*p* < 0.01) was observed with 20 ppm bromoform inclusion, but not with 0 and 9.5 ppm inclusion levels. Experiment 3 (30 d finishing trial) tested bromoform effects on feeder calves. DMI (*p* = 0.53), average daily gain (*p* = 0.55), and gain:feed (*p* = 0.82) were not influenced by bromoform inclusion. Bromoform residues were undetectable in liver, kidney, adipose, and muscle samples collected at harvest. These experiments demonstrated that cattle fed PKBP experience short-term reductions in CH_4_ without tissue accumulation of bromoform and without evidence of effects on animal growth or feed consumption.

## 1. Introduction

Methane is a potent greenhouse gas. According to the IPCC, increases in atmospheric CH_4_ concentrations since the industrial revolution have contributed to about 0.5 °C of the approximate 1 °C observed global warming over that time period [1]. Livestock production is considered to be responsible for about 1/3 of the global annual anthropogenic CH_4_ production, primarily through enteric methanogenesis in ruminant animals [2]. While a variety of mitigation strategies have been tested to minimize the amount of CH_4_ associated with ruminants, few have been as effective as red seaweed, or kelp, (i.e., genus *Asparagopsis*). The addition of *Asparagopsis taxiformis* has been reported to decrease enteric CH_4_ production by 50–99% when included at 0.25–1.0% of DM in cattle rations [3,4]. The observed abatement in CH_4_ emissions observed with the inclusion of kelp has been attributed to synthesis of halogenated methane analogs, such as bromoform.

Bromoform is a competitive inhibitor of methyl coenzyme M reductase (MCR), an essential step in methanogenesis [5]. When MCR is inhibited, the formation of CH_4_ is blocked, thereby halting methanogen metabolism and inducing death of methanogenic archaea [5]. Due to the slow regeneration time of methanogens (i.e., the target of bromoform), it is likely that the reduction in CH_4_ is temporarily sustained after cessation of supplementation. If supplementing with bromoform-containing feeds is determined to maintain lowered CH_4_ levels, costs and level of exposure associated with administering bromoform for the purposes of CH_4_ abatement may be decreased.

Chronic exposure to bromoform has been associated with increased risk of health complications [6]. Bromoform has been implicated in changes to hepatic and renal function in rats [7,8] (as reported by [6]) as well as being listed as a possible carcinogen by the CDC, particularly when exposure occurs orally. Thus, strict levels have been established for items intended for human consumption [6]. Given the significant amount of evidence suggesting negative health effects in mice and rats with bromoform exposure, the EPA recommends that drinking water should contain no more than 0.7 ppm of bromoform, and OSHA established limits for the amount that workers can be exposed in air during an 8-h work day (0.5 ppm; [6]). These concerns with bromoform have led others to investigate possible modes of excretion or retention by livestock supplemented with this kelp-containing halogenated methane analogue, which may threaten the health of consumers. Roque et al. [3] observed no detectible bromoform residues in longissimus dorsi samples from cattle consuming *A. taxiformis* for 21 weeks. Stefenoni et al. [4] did not find bromoform above the limit of detection in milk from dairy cows. Although these two studies provided novel data regarding the potential for meat and milk contamination, to our knowledge the literature does not contain reports examining organ or fat tissue samples for bromoform residues.

In addition to potential health implications, supplementing bromoform in rations may impact growth. During 147 d of supplementation to cattle, Roque et al. [3] observed that dry matter intake (DMI) tended to decrease by 8% when *A. taxiformis* was included in rations to supply 39.1 ppm bromoform and by 14% with 78.2 ppm bromoform. Stefenoni et al. [4] observed a similar decrease of 7.8% in DMI by dairy cows when *A. taxiformis* was included at 0.5% DM (bromoform concentration not reported) for 28 d. Methane losses are estimated to vary among animals from 2–12% of gross energy intake, with increased intake [9] and digestibility [10] noted to mitigate these losses to a certain extent. Because both factors (i.e., intake and digestibility) are direct contributors to animal performance, body weight gain and growth efficiency measures during bromoform supplementation can be used as proxies for methane and gross energy losses when direct measures of methane production are unavailable.

Thus, the objectives of these experiments were multifold. Experiment 1 sought to record the effects of a proprietary kelp blend product (PKBP) containing metabolites, including bromoform, on lowering enteric methane production in growing calves. Experiment 2 was designed to determine the duration of the inhibitory effects of bromoform on CH_4_ production and yield in growing steers. Experiment 3 investigated the potential effects of PKBP on DMI, average daily gain (ADG), gain:feed ratio (i.e., growth efficiency; G:F), and concentration of bromoform in the liver, kidney, subcutaneous fat, and *Longissimus dorsi* muscle tissue.

## 2. Materials and Methods

### 2.1. Ethics Statement

These experiments were conducted at the University of Kentucky C. Oran Little Beef Research Unit, following a protocol (Protocol #2022-4019) approved by the Institutional Animal Care and Use Committee of the University of Kentucky. Furthermore, Experiments 1 and 2 were conducted in an environmentally controlled, thermoneutral environment. Experiment 3 was conducted in outdoor pens with partial shade.

### 2.2. Experiment 1

#### 2.2.1. Animal Management and Housing

Twelve Holstein steers (161 ± 8.5 kg BW) were used in a 14 d randomized complete block design experiment to test the hypothesis that supplementing PKBP (as a source of bromoform) would reduce enteric methane production. Prior to beginning the experiment, steers were vaccinated (Titanium™ 5, Elanco Animal Health, Greenfield, IN, USA; Once PMH^®^ SQ and Vision^®^ CD-T, Merck Animal Health, Rahway, NJ, USA; Ultrabac^®^ 7/Somubac^®^, Zoetis Inc., Kalamazoo, MI, USA), treated with parasiticide (Eprizero™ Pour-on, Norbrook, Lenexa, KS, USA), and backgrounded on a corn silage-based diet for a minimum of 14 d. Steers were housed indoors under thermoneutral conditions (22 °C) individually in 3 m × 3 m stalls, given ad libitum access to water via continuous water basins, and fed treatment diets beginning 11 d prior to the methane measurement period. A 14:10 light: dark cycle was established with lights turning on at 0600 h and off at 2000 h each day. During the methane measurement period, steers were moved within the same facility to metabolism stalls (1.25 m × 2 m) fitted with indirect calorimetry headboxes for 72 h of semi-continuous measures of gas production. Animals received daily rations at 0800 h, which were formulated to provide 1.5 × NE_m_ of growing Holstein steers, along with corresponding levels of minerals and vitamins [11].

#### 2.2.2. Experimental Design

Steers were blocked (*n* = 4 per block) by weight and randomly assigned to one of two treatments (*n* = 6 per treatment with 2 animals per treatment within each block). With four headboxes available, three collection periods were necessary for indirect calorimetry measures. Thus, each of the three blocks was assigned to a different collection period, such that blocks accounted for random effects associated with both weight and collection period. Dietary treatments consisted of a corn silage-based diet (Table 1) that was supplemented at a rate of 5% of the total DM with either a ground corn carrier (control) or a ground corn carrier including PKBP. Given variation in assayed bromoform concentrations in PKBP, the PKBP was included in the treatment supplement at a rate that supplied 52.5 ± 10.5 ppm of bromoform in the total ration dry matter beginning 11 d prior to the start of the respiratory gas collection period. Bromoform concentration of the PKBP was provided by the supplier as determined using the methods of Paul et al. [12].

The test treatment supplement was prepared by mixing PKBP with a ground corn carrier in a commercial stainless-steel chopper (Mandeville Company, Inc., Minneapolis, MN, USA) for 10 min followed by 10 s of pulse-blending (Waring MX1000XTX Extreme, Waring Commercial, McConnellsburg, PA, USA) to produce a uniform distribution of PKBP throughout the supplement. The control supplement containing only ground corn was subjected to identical procedures to ensure that minimal differences in particle size existed between the two supplements. Supplements were prepared prior to each collection period and stored in sealed containers at 5 °C until fed (Rubbermaid Commercial Products, Sarasota Spring, NY, USA). Each supplement was top-dressed on the corn silage-based ration and hand mixed in the feed bunk. The basal diet was subsampled daily and composited weekly for dry matter analysis (55 °C, forced air oven). Similarly, any feed refusals were weighed, sampled, dried, and included in the calculation of DMI.

#### 2.2.3. Enteric Gas Collection 

Enteric methane production, as well as CO_2_ production and O_2_ consumption, were measured by confining the animals in metabolism stalls fitted with stainless-steel headbox style indirect respiratory chambers. As previously described by Koontz et al. [13], each respiration chamber (0.90 m × 1.5 m × 0.60 m) was composed of stainless-steel with three plexiglass windows (0.30 m × 0.60 m on sides and 0.90 m × 0.60 m on rear) and equipped with a shroud to be placed at the base of the animal’s neck [13]. Each respiration chamber was fitted with a continuous water basin to allow for ad libitum intake of water and a door at the rear of the chamber (0.90 m × 0.60 m) to allow for placing feed in the feeder. Each chamber was built around a stall designed for intensive metabolic studies including an adjustable track to allow for extension of the bedding area (1.84–2.44 m × 1.68 m), which was covered with a rubber mat. Each respiration chamber was also equipped with an air conditioning unit to maintain ambient temperature (21 °C) and relative humidity (35%). 

Prior to use, the zero point of O_2_, CO_2_, and CH_4_ gas analyzers was calibrated with pure N_2_ gas (American Welding & Gas, Lexington, KY, USA) and the span point of each gas analyzer was calibrated with a custom calibration gas (American Welding & Gas, Lexington, KY, USA) containing 19.90% O_2_, 0.70% CO_2_, and 0.065% CH_4_ that was accurate within 1% tolerance. Each respiration chamber was validated by combusting propane over a 120 min period and determining the mass of propane combusted relative to the amount of CO_2_ and O_2_ measured within the respiration chamber. 

Air flow from each respiration chamber was measured using HFM-200 mass flow meters (Teledyne Hastings Instruments, Hampton, VA, USA) with vertically imposed LS-4F laminar flow elements (Teledyne Hastings Instruments, Hampton, VA, USA) and maintained during measurement periods via flow controller (Flow Max XL; Columbus Instruments, Columbus, OH, USA) at 300 L/min. Air from the chambers passed through a 10-channel expansion interface and sample pump (0.5 L/min). Prior to entering analyzers, sample gases passed through sample drying tubes filled with indicator desiccant (W.A. Hammond Drierite Co., Ltd., Xenia, OH, USA). Samples were then analyzed for O_2_ via paramagnetic sensor (Columbus Instruments, Columbus, OH, USA), whereas CO_2_ (Columbus Instruments, Columbus, OH, USA) and CH_4_ (VIA-510; Horiba Ltd., Kyoto, Japan) were measured via infrared sensors. Data were communicated to a computer interface through a CI-Bus Serial Interface (Columbus Instruments, Columbus, OH, USA) at 4 min intervals to measure background and chamber gas concentrations, with measurements alternating among the 4 headboxes in sequence such that respiration gases from each animal were analyzed every 16 min. Data were recorded via computer interface using Oxymax software (version 4.8.5; Columbus Instruments, Columbus, OH, USA). To ensure animal safety during gas measurement periods, the computer interface and animals were monitored continuously to ensure CO_2_ concentrations remained at 0.4–0.7% and power supply remained available. Following collection, O_2_ consumption, CO_2_ production and CH_4_ production were calculated as total volume of gas consumed/produced during 24 h increments between feedings and were averaged over the 72 h period. Data obtained during intervals in which the headbox was open (i.e., for feeding) were excluded from calculations.

### 2.3. Experiment 2

#### 2.3.1. Animal Management and Respiratory Gas Measurement

**Figure 1:**
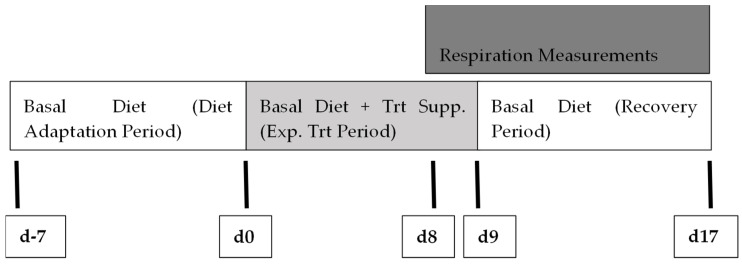
Experiment 2 Timeline for Holstein steers’ adaptation to and subsequent change from a corn silage-based diet supplemented with either control (*n* = 2), 9.5 ppm (*n* = 2), or 20 ppm bromoform (*n* = 2). PKBP inclusion formulated to provide bromoform at the targeted levels for each treatment. At the end of the treatment period, steers were removed from individual pens and placed in headbox calorimeters for collection of respiratory gases to compare methane production differences among treatments. All steers were placed on the control treatment for the duration of the study after the first 24 h collection of respiratory gases to monitor recovery of enteric methane production following PKBP supplementation.

Holstein steers (*n* = 6; 342 ± 10.5 kg BW) were vaccinated as in Experiment 1, treated with parasiticide (Dectomax^®^ Injectable, Zoetis Inc., Kalamazoo, MI, USA), and individually penned (3.0 m × 3.7 m pens) indoors under thermoneutral (22 °C) conditions. Each pen was equipped with a feed bunk and automatic waterer which allowed for ad libitum access to water for a 17 d study including a respiratory gas collection period (Figure 1). During d-7 through d-1, steers were adapted to their basal diet plus the control supplement. On d0 to d8, steers were fed the basal diet with their experimental treatments (0, 9.5 ± 1.5, or 20 ± 3 ppm bromoform). On d8, after consuming their daily allotment of feed, steers were transferred into headbox style respiratory calorimeters for adaptation to the new environment prior to the collection period (d 9–17). Beginning on d10 and lasting the duration of the study, all steers received the control supplement for evaluating recovery of enteric methane production following a period of bromoform consumption (Figure 1).

Methane and CO_2_ production and O_2_ consumption were measured as described for Experiment 1, with the addendum that air flow was increased to and maintained at 600 L/min to account for the greater body mass of steers in Experiment 2. Measurement intervals were also decreased to 12 min, as mechanical issues prevented the use of one respiration chamber.

#### 2.3.2. Experimental Design

As in Experiment 1, steers were blocked by weight and randomly assigned within each block to one of three treatments (*n* = 3 per treatment). Two collection periods were necessary for indirect calorimetry measures due to the availability of only three headboxes, with each collection period associated with a different block. Thus, blocks were used to account for random effects associated with both weight and collection period. Figure 1 is a timeline of the experimental period for Experiment 2. From d-7 to d0 steers received the basal diet and control supplement; from d1 to d 9 steers received the basal diet plus their appropriate treatment supplement, supplied at 5% of the total DM. Beginning on d10, and through the remainder of the experiment, all steers were switched back to the control supplement to measure residual effects of the treatment. Supplement was prepared prior to the beginning of each block by mixing PKBP with a ground corn and distillers dried grain carrier in a commercial stainless-steel chopper (Mandeville Company, Inc., Minneapolis, MN, USA) for 10 min until uniform distribution of PKBP was achieved. Following mixing, top dress was stored at −20 °C in plastic containers (Rubbermaid Commercial Products, Sarasota Spring, NY, USA) with a plastic cover and snap-on lid. This difference in methodology from Experiment 1 was necessitated by changes in PKBP manufacturing.

A corn silage-based diet (Table 1) was top dressed with the assigned treatment supplement and mixed thoroughly. Mixed rations were fed once daily at 0800 to provide 1.5 × NE_m_ to each animal. The diet ingredients were subsampled daily and composited weekly for DM analysis (55 °C forced air oven). For each collection, a 200 g sample (in duplicate) was collected, weighed, and dried for DM analysis. Feed refusals were collected daily, weighed, subsampled (duplicate 100–200 g samples) and dried for DM analysis. Dry matter was determined by weighing samples daily until sample weight remained constant for two consecutive days. Values for refusals were used in calculations for DMI. Animals were weighed on d-7 and d-1, with d-1 weights used to determine intake for each steer during the experimental period.

### 2.4. Experiment 3

#### 2.4.1. Animal Management

Angus steers (*n* = 12; 424 ± 12.5 kg BW) were housed outdoors in individually covered outdoor pens (4.8 m × 14.6 m) equipped with concrete feed bunks and automatic waterers that allowed for ad libitum access to water for a 30 d feeding study. For the duration of the experiment, steers were fed a corn silage-based diet (Table 1) once daily at 0800. Feed offered was adjusted twice weekly to provide ad libitum intake with minimal residuals. Prior to the beginning of the study, animals were adapted to ad libitum intake of an 80% concentrate basal diet for a minimum of 14 d.

#### 2.4.2. Experimental Design

Steers were blocked by weight and assigned randomly to one of three dietary treatments (*n* = 4 steers/treatment). Supplements were composed of a ground corn carrier with distillers dried grains (control). PKBP treatments were attained by mixing the control treatment with PKBP. Each supplement was top dressed at a rate of 5% of the total ration DM, which supplied 0, 9.5 ± 1.5, or 20 ± 3 ppm bromoform in the total ration. PKBP was prepared and stored as described for Experiment 2. Individual ingredients of the basal diet were sampled (500 g) weekly and analyzed for DM by weighing duplicate 200–250 g samples, which were dried in a 55 °C forced air oven until weight of sample remained consistent for two consecutive days. Weekly DM determinations were used to adjust dietary ingredients and to calculate dry matter intake (DMI). The amount of feed offered was adjusted twice weekly to provide ad libitum intake with minimal refusals. Any feed refusals were collected weekly, weighed (duplicate 200–250 g samples) and dried at 55 °C in a forced air oven for DM determination. Steers were weighed prior to beginning the feeding period (d 0) and prior to transport to the University of Kentucky Meat Sciences Laboratory abattoir (Lexington, KY, USA; d 30). Start dates for the feeding period were staggered by block across a 19 d period to provide each steer a 30 d feeding period. Such scheduling was required to allow for processing and “tanked” carcass storage limitations, and scheduling conflicts at the abattoir.

Upon completion of each feeding period, steers were transported to the University of Kentucky abattoir where they were humanely harvested under USDA inspection using a captive-bolt followed by exsanguination. Immediately following the removal of the hide and separation of the carcass and organs, approximately 1 g (wet weight) samples from the liver, kidney, subcutaneous fat, and longissimus dorsi muscle at the 12th/13th rib interface were harvested using a biopsy needle (Bergstrӧm-Stille, Torshӓlla, Sweden). Tissues were placed into vials labeled with animal ID and tissue type and immediately frozen in liquid nitrogen until all samples were harvested. Once collection was completed, samples were stored at −80 °C until samples had been collected from all animals. After tissues were harvested from the final group of steers, samples were shipped to Bigelow Analytical Services (BAS: East Boothbay, ME, USA) for determination of bromoform concentration. Methods developed by Paul et al. [12], with modifications developed by BAS, were used for determination of bromoform in harvested tissues. Once samples arrived at BAS, they were immediately placed in a freezer (−80 °C) until extractions took place. In summary, samples were weighed and extracted in MeOH with 4 µg/mL naphthalene as an internal standard. The extraction volume was approximately 0.5 mL MeOH extractant/g sample, a ratio established following extensive testing for extraction efficiency [12]. The sample and MeOH extractant were then bead-beaten for 15 min (Retsch Mixer Mill MM40, Haan, Germany) at 30 Hz, with 2.0 mm zirconia oxide beads per sample. Next, samples were centrifuged for 5 min at 15,000 rpm [12]. Samples were then transferred via glass syringe into GC-MS vials. MeOH extracts were then quantified using GC-MS (Shimadzu QP2010 GC-MS, 30-m RTX 502.2 column, Kyoto, Japan). Injections of 1 µL were performed with a split ratio of 20 with an inlet pressure of 8 psi. The injection port temperature was set to 200 °C and the interface temperature was 220 °C. A selected ion monitoring mode (SIM) was used for ions *m/z* 173 and 128, with the temperature program starting at 65 °C and immediately increasing to 220 °C at 30 °C/min with a 3 min hold at 220 °C (total run time: 8.17 min). All other steps of the original method [12] were used for finalizing sample bromoform quantification. Sample limits of detection were established for each of the tissues prior to quantification of bromoform and are listed in Table 8.

### 2.5. Statistical Analysis

Experiment 1 data were analyzed as a randomized complete block design using the MIXED procedure of SAS (9.4, Cary, NC, USA) with the Kenward-Roger 2 option selected as the denominator degrees-of-freedom method. Treatment and block were included as fixed variables. Oxygen consumption and CO_2_ and CH_4_ production were totaled over each 24 h period and averages for each animal were included as dependent variables. Following collection from all blocks, it was determined that CH_4_ concentrations in expired air were below detection limits in the treatment group. To account for this, maximal CH_4_ production was estimated for this group using the lowest CH_4_ concentration (50 ppm) in the exhaust air of control animals. Because of the absence of measurable variance for the treatment group, the variance observed for the control group was used across treatments. Typical ANOVA (as with Proc Mixed in SAS) assumes homogeneous error variance among treatments. With all values below detection limits for CH_4_ concentrations for one treatment group, this would yield an artificially large F-value because the pooled error term would effectively be averaged between one treatment with measurable variance and one treatment with zero variance, yielding an artificially small value for the denominator in the calculation of the F-value. With the RCBD used in this experiment, the SSE calculated in SAS Proc Mixed was actually determined based on 3 df (as opposed to 8 df assumed in the Proc Mixed ANOVA), so a corrected MSE was determined as SSE/3, and this value was used in the denominator for the calculation of the corrected F-value for treatment. The probability of a greater F-value was then generated using the F.DIST.RT function of Microsoft Excel (v. 2405), with 1 numerator df and 8 denominator df as appropriate for the RCBD model used). This approach is consistent with an assumption of homogeneous error, despite that fact that error variance could not be determined for the treatment in which all values were below detection limits, and yields a more conservative (and accurate) estimate of the *p*-value than would be obtained without such correction. Dry matter intake was analyzed separately within the treatment adaptation period and gas collection period. One steer assigned to the PKBP treatment was removed from analyses due to illness and low DMI. Based on the subsequent observation and death two weeks after the experiment, the illness was not considered related to the experimental treatment. Significance was set at *p* ≤ 0.05.

Prior to analyses, data for each timepoint collected during the respiratory gas measurement period of Experiment 2 were screened for anomalous levels, as there were multiple instances of steers exiting the respiration chambers, resulting in inaccurate data points. These anomalous values were deemed to be outliers based on O_2_ and CO_2_ percentages of measured air (e.g., the delta values between intake and exhausted air samples), and accounted for 94 of the 5173 data points collected across all animals. These timepoints were removed from the dataset and the number of minutes associated with the removed data points was subtracted from 1440 (minutes in a 24 h period) to determine the total number of minutes associated with the included data (TIME). Total consumption of O_2_ and production of CO_2_ and CH_4_ was summed for each day, divided by TIME, and multiplied by 1440 min to attain the total amount of each gas consumed or produced during the 24 h period. Data were then analyzed using the MIXED procedure of SAS (9.4) with a repeated measures model to account for autocorrelation within animal measurements. A first order autoregressive correlation structure was assumed. Linear, quadratic, and cubic contrasts were included for day effects (or, when day × treatment effects were significant, for day within treatment), and linear and quadratic contrasts were included for treatment. Treatment means on specific days were also separated using Fisher’s LSDs through use of the LSMEANS diff option. Response variables included DMI, CH_4_, O_2_, and CO_2_. Significance was set at *p* ≤ 0.05.

Following initiation of Experiment 3, it was determined that there was insufficient PKBP to support ad libitum intake of the treatments throughout the 30 d feeding period due to greater than expected intakes by the steers. Thus, a decision was made to maintain the 30 d feeding period and reduce the number of observations made for the two PKBP treatments. Additionally, one animal was removed from the dataset following detection of a significant abscess in the abdominal cavity during harvest, which was determined to be the cause of decreased dry matter intake and absence of growth over the 30 d period and, more importantly, unrelated to treatment. Cumulatively, this resulted in an unequal number of observations between treatments (*n* = 4 for control, *n* = 3 for 9.5 ppm bromoform, and *n* = 2 for 20 ppm bromoform). Data were analyzed using the MIXED procedure of SAS (9.4). Response variables included DMI, average daily gain (ADG), growth efficiency (ADG/DMI), and concentration of bromoform in liver, kidney, subcutaneous fat, and longissimus dorsi muscle tissue. Least square means were separated using orthogonal linear and quadratic contrasts and significance was set at *p* ≤ 0.05.

## 3. Results

### 3.1. Experiment 1

#### 3.1.1. Intake

The amount of feed offered was limited to 1.5 × NE_m_ of growing steers (Table 2). Accordingly, dry matter consumption was nearly complete and exceeded 2.0% of body weight across treatments. During the gas collection period, bromoform-treated animals refused up to 0.33 kg of offered feed on 2 days (*n* = 2) and up to 0.36 kg on 3 days (*n* = 1). With DMI expressed as a function of body weight, there were no significant differences between treatments (*p* ≥ 0.11) during either time period.

#### 3.1.2. Enteric Gas Production

**Figure 2:**
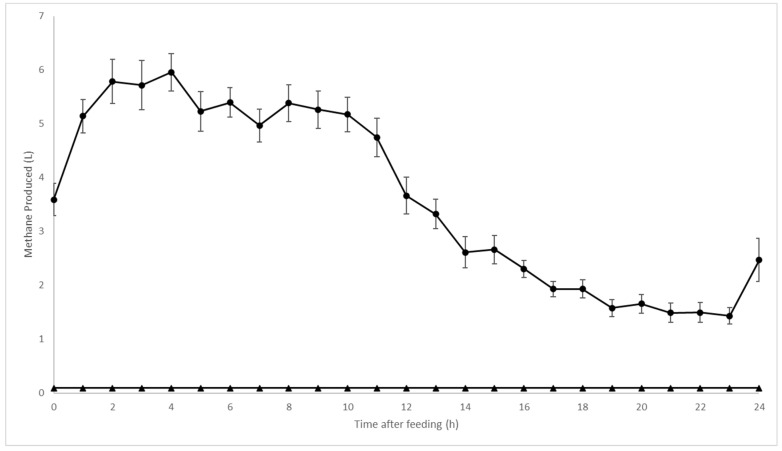
Experiment 1 methane production during 24 h measurements of steers consuming corn silage-based diet supplemented with either control (*n* = 6) or 52.5 ppm bromoform (*n* = 5). Methane concentration in exhaust air, and thus production, was below detectable limits for all time points in steers supplemented with PKBP. ● = control treatment; ▲ = 52.5 ppm bromoform treatment.

Methane production was significantly reduced (*p* < 0.001) by PKBP treatment (Table 3). Steers consuming the control supplement exhibited the expected post-prandial increase in methane production (Figure 2), which resulted in a mean production of 88.7 L/d. In contrast, the CH_4_ concentrations of exhaust air from steers consuming the PKBP treatment were below detectable levels at all measured time points throughout the 72 h measurement period. For the purpose of statistical comparison, the lowest detectable CH_4_ concentration was set at 2.2 L/d for the PKBP group based on the set flow rate and minimum level of detection for the analyzers. Methane yield of control treatment animals averaged 18.96 g/kg of DMI compared to a maximum of 0.49 g/kg of DMI for PKBP treatment animals. Oxygen consumption (*p* ≥ 0.20) and CO_2_ production (*p* ≥ 0.28) did not differ between treatments or blocks.

### 3.2. Experiment 2

#### 3.2.1. Dry Matter Intake

The amount of feed offered was limited to 1.5 × NE_m_ requirement of growing steers [11]. All feed was consumed throughout the entire adaptation and respiratory gas collection periods.

#### 3.2.2. Methane and Respiratory Gases

**Figure 3:**
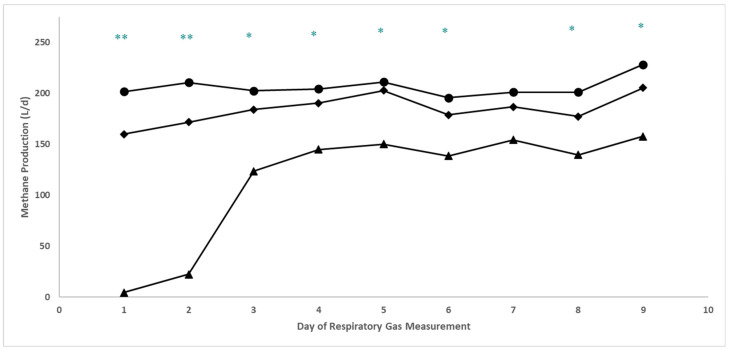
CH_4_ production (L/d) in Holstein steers (*n* = 6) during and following PKBP supplementation (Experiment 2). Daily CH_4_ production of steers consuming corn silage-based diet supplemented with either control (*n* = 2), 9.5 ppm (*n* = 2), or 20 ppm (*n* = 2) bromoform dietary inclusion (d 1) or control supplement only (d 2–9; post-supplementation). A cubic response for the treatment (day) was observed for the 20 ppm bromoform treatment (*p* = 0.02), but no orthogonal polynomial contrasts were significant (*p* ≥ 0.11) for the 9.5 ppm bromoform and control treatments. When expressed on a L/d basis, methane treatment means for steers given the 20 ppm treatment were lower than those on the other two treatments throughout the recovery period, except for d7 and d8, when no difference was detected between 20 ppm and 9.5 ppm bromoform treatments. ♦ = control treatment; ● = 9.5 ppm bromoform treatment; ▲ = 20 ppm bromoform treatment; ** = quadratic response across treatments; * = linear response across treatments.

**Figure 4:**
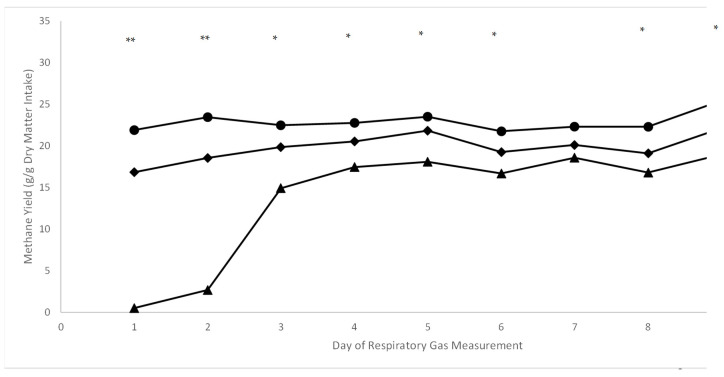
CH_4_ yield (g/g dry matter intake) in Holstein steers (*n* = 6) during and following PKBP supplementation (Experiment 2). Daily CH_4_ yield of steers consuming corn silage-based diet supplemented with either control (*n* = 2), 9.5 ppm (*n* = 2), or 20 ppm (*n* = 2) bromoform dietary inclusion (d 1) or control supplement only (d 2–9; post-supplementation). A cubic response for the treatment x day was observed for the 20 ppm bromoform treatment (*p* = 0.01), but no orthogonal polynomial contrasts were significant (*p* ≥ 0.09) for the 9.5 ppm bromoform and control treatments. When expressed on a g/g dry matter intake basis, methane treatment means for steers given the 20 ppm treatment were lower than those on the control treatment throughout the recovery period, and lower than those on the 9.5 ppm bromoform treatment on d1 to d3. ● = control treatment; ♦ = 9.5 ppm bromoform treatment; ▲ = 20 ppm bromoform treatment; ** = quadratic response across treatments; * = linear response across treatments.

**Figure 5:**
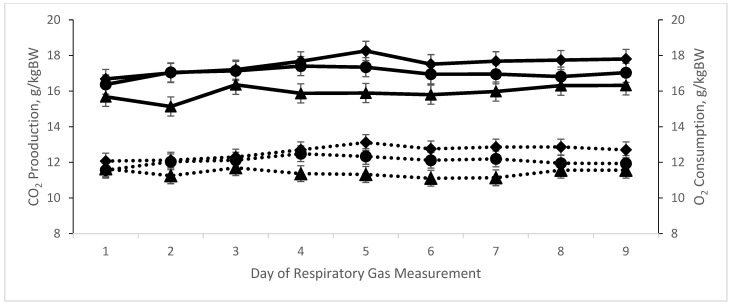
CO_2_ production and O_2_ consumption expressed on a bodyweight basis (g/kg BW) in Holstein steers (*n* = 6) during and following PKBP supplementation (Experiment 2). Steers consuming corn silage-based diet supplemented with either control (*n* = 2), 9.5 ppm (*n* = 2), or 20 ppm (*n* = 2) bromoform dietary inclusion (d 1) or control supplement only (d 2–9; post-supplementation). A quadratic response across treatments was observed for both O_2_ (g/kgBW; *p* < 0.01) and CO_2_ (g/kgBW; *p* < 0.01) such that steers receiving the 9.5 ppm bromoform treatment diet had greater O_2_ consumption and CO_2_ production than steers receiving the 20 ppm treatment, but were not different from control steers’ usage and production of these gases. A linear effect of treatment across days was detected for 20 ppm bromoform-treated steers, but not the other two treatments, for O_2_ consumption, but this phenomena appears to largely be an effect of greater O_2_ usage on d5 compared with d1 and 2. Dotted line = O_2_; solid line = CO_2_; ● = control treatment; ♦ = 9.5 ppm bromoform treatment; ▲ = 20 ppm bromoform treatment.

Respiratory gas data are presented in Table 4 and Table 5, and Figure 3, Figure 4 and Figure 5. A treatment × day interaction (Table 4; *p* ≤ 0.01) was observed for both enteric CH_4_ production (L/d; Table 4 and Figure 3) and yield (g/g DMI; Table 4 and Figure 4). Steers consuming 20 ppm bromoform had a cubic response (*p* = 0.02) in CH_4_ production over time, with lower production of this GHG maintained throughout the 9 d measurement period. A similar cubic response (*p* = 0.01) for the high-bromoform-treated steers was observed when CH_4_ was calculated on a yield basis. In contrast, no linear, quadratic, or cubic responses across days were detected for CH_4_ production or yield for the 9.5 ppm bromoform and control treatments. Interestingly, regardless of how CH_4_ was expressed (i.e., L/d, g/g DMI, g/kg BW), when examined within days across treatments, a negative quadratic response was detected on d1 and 2 (*p* ≤ 0.05; Table 5), and a linear decrease across treatments (*p* ≤ 0.05; Table 5) was observed for all other days except d7. O_2_ consumption (L/d) and CO_2_ production both responded quadratically (*p* ≤ 0.02; Table 6) to increasing bromoform, with slight increases at 9.5 ppm and more substantial decreases at 20 ppm inclusion levels. A linear effect of treatment across days was detected for 20 ppm bromoform-treated steers, but not the other two treatments, for O_2_ consumption. However, this appears to largely be an effect of greater O_2_ usage on d5 compared with d1 and 2 (Figure 5).

### 3.3. Experiment 3

#### Intake, Growth Performance, and Bromoform Residues

Dry matter intake linearly decreased (*p* < 0.01) with increasing bromoform inclusion level (Table 7). However, when DMI was expressed on a body weight basis, no effect (*p* ≥ 0.23) of treatment was detected. Neither average daily gain (kg/d) nor G:F (g/kg) were affected by treatment (Table 7; *p* ≥ 0.41 and *p* ≥ 0.60). Most importantly, following the 30 d feeding period, no bromoform residues were detected in any of the tissues (liver, kidney, muscle, or adipose) across treatments (Table 8). Limits of detection ranged from 3.7 to 9.1 µg/kg.

## 4. Discussion

### 4.1. Intake and Growth Performance

Given the limitations of these studies for assessing intake, results provide some weak evidence indicating potential for intake depression with increasing levels of dietary bromoform. The few feed refusals in Experiment 1 were confined to animals consuming bromoform, and in Experiment 3, where statistical power was limited for this measure, numerical trends were aligned with a linear decrease in intake (as a % of BW) with increasing bromoform inclusion. In Experiments 1 and 2, the amount of feed offered was limited to 1.5 × NE_m_ of growing steers. Accordingly, dry matter consumption was nearly complete and exceeded 2.0% of body weight across treatments. In Experiment 3, ad libitum dry matter consumption as a function of BW was not affected by treatment despite a numerical reduction in intake of 4% and 11% for the 9.5 and 20 ppm treatments, respectively. The performance data in this study were largely collected for content of measured tissue residues and represent limited observations as compared to typical performance studies and therefore should be interpreted with some caution. Kinley et al. [14] did not observe effects on DMI when beef steers consuming a high grain TMR were supplemented with kelp to provide 0, 3.6, 7.2, and 14.2 ppm (DM basis) bromoform. Other studies have shown potential for increasing bromoform to be associated with decreased DMI. Stefenoni et al. [4] observed a quadratic response in DMI with increasing *A. taxiformis* inclusion levels, with increased or no change in intake at 0.25% DM inclusion, but decreased intake when supplementing at 0.5% and 0.75% DM when compared with non-supplemented animals. The bromoform content of that study was not defined; thus, it is difficult to make conclusions on the differences in reported intake. Roque et al. [15] observed 11% and 38% decreases in DMI with 6.6 and 13.2 ppm bromoform from *Asparagopsis armata* additions to dairy cow rations.

The potential to recapture the energy that would have been lost in the form of CH_4_ is a major goal of mitigation studies. However, detrimental effects of these mitigation strategies on DMI could eliminate the potential benefits of inclusion if growth performance is negatively impacted. The absence of differences among treatments for ADG in the current experiment is in agreement with Roque et al. [15], who reported no changes in ADG across treatments when beef cattle were supplemented with either 19.6 or 39.1 ppm bromoform. However, G:F during Experiment 3 was unaffected by treatment, whereas Roque et al. [15] observed a significant increase in growth efficiency in response to increasing kelp concentration in the ration. This difference in growth efficiency effects may relate to the number of observations or days on feed, both of which were greater in Roque et al. [15].

### 4.2. Enteric Gas Production during Supplementation

Methane yield of control treatment animals averaged 18.96 g/kg DMI compared to a maximum of 0.49 g/kg DMI for PKBP treatment animals. Vucko et al. [16] reported a strong, negative correlation between in vitro CH_4_ production and bromoform concentration (r^2^ = −0.965). Roque et al. [3] compared varying forage-to-concentrate ratios with the inclusion of bromoform (0.0, 18.4, or 36.8 ppm) in the diet. When cattle were consuming a low-forage diet supplemented with kelp, CH_4_ yield decreased from 12.4 g/kg DMI in control animals to 3.75 and 2.50 g/kg DMI in 18.4 and 36.8 ppm bromoform supplemented animals, respectively [3]. The authors of that study observed a 50.6 and 74.9% reduction in methane production for 18.4 and 36.8 ppm bromoform diet inclusion, respectively, which is less than the 97% reduction observed for animals receiving 52.5 ppm bromoform in Experiment 1 and 20 ppm bromoform in Experiment 2 of the current study. Kinley et al. [14] observed beef steers consuming a high grain diet mixed with *A. taxiformis* at a rate to supply 14.2 ppm bromoform (DM basis) generated 98% less CH_4_. The supplementation period used for that study was 90 d, which may have allowed for lower bromoform inclusion to achieve comparable results to Experiments 1 and 2.

Bromoform, the active component in kelp, binds methyl coenzyme M reductase (MCR), which is an essential step of methanogen metabolism [5]. By binding the enzyme, catalysis required to form CH_4_ cannot be completed, which decreases CH_4_ production and methanogen presence in the rumen [5]. Alterations in the microbial structure in the rumen can potentially cause detrimental changes to whole animal metabolism. As we observed similarities between treatments for DMI, and thus energy intake, as well as no differences in the respiratory quotient among treatments, oxidative metabolism appears to have remained unaffected by PKBP supplementation.

### 4.3. Enteric Methane and Respiratory Gas Post-Supplementation Recovery

During Experiment 2, d 1 of the gas measurement period overlapped with the final day of PKBP supplementation, whereas on d 2 through d 9 all steers received the control supplement. Therefore, the initial day of the measurement period represented the concurrent impact of bromoform supplementation on CH_4_, and the remaining time points reflected residual, post-supplementation effects. Although the bromoform concentration used was lower than in Experiment 1 (20 ppm vs. 52.5 ppm), 20 ppm inclusion for 9 d was sufficient to eradicate CH_4_ production and to suppress production (compared with 0 ppm bromoform inclusion levels) for up to 9 d after supplementation ended. Additionally, the reduced methane (relative to control animals) with 9.5 ppm bromoform during the first 2 d of the recovery period indicates this dosage level may be sufficient to suppress methane production when provided on a daily basis.

To our knowledge, these are the first data available on recovery of CH_4_ following cessation of bromoform supplementation. Inhibition of MCR interrupts the metabolism of methanogens, causing cell death [17]. Methanogens are also known to be slow growing. Thus, substantial reductions in ruminal methanogen numbers should dictate a long recovery time [18]. 3-Nitrooxypropanol (3-NOP), another anti-methanogenic compound, is believed to work through a mechanism similar to that of bromoform via targeting MCR [19]. Romero-Perez et al. [20] investigated the potential of 3-NOP to sustain mitigation following 112 d of 3-NOP supplementation (2 g/d) to a total mixed ration (60% barley silage, 35% barley grain, 5% vitamin–mineral supplement). At the end of 112 d of supplementation, CH_4_ production was reduced 59.2%, although there were no residual effects of 3-NOP during the recovery period [20]. These results differ from the findings of the current experiment, although the reason for the differences in findings is unclear. The data from the current study suggest PKBP supplementation may not be needed daily when provided at ≥20 ppm of the ration. Thus, future research should focus on developing a protocol of repeated delivery of PKBP that does not require daily supplementation. Additionally, these studies should address scenarios in which cattle are commingled. Such environments can contribute to microbial exposure, which could allow for re-inoculation of the rumen and thus impact duration of residual effects.

Reductions in O_2_ and CO_2_ are indicative of reduced heat production. Whether this is a function of reduced digestibility or changes in metabolism is unclear. However, in preliminary unpublished in vitro studies from our laboratory, VFA concentrations were not affected by the inclusion of PKBP, suggesting absence of effects on digestibility. These findings concur with published studies in which kelp inclusion did not affect VFA concentrations [21,22]. The observed differences in CO_2_ production in this study were not consistent with Roque et al. [3], who observed no changes in CO_2_ production (g/d) or intensity (g/kg ADG) when supplementing bromoform at 39.1 ppm (with *A. taxiformis*) to cattle consuming high-, medium-, or low-forage diets. Differences between the findings of that study and Experiment 2 are likely due to the reported decreases in DMI of Roque [23] that were not observed in this study. Without concurrent changes in DMI, CO_2_ and CH_4_ production would be expected to have an inverse relationship, but we instead observed a concurrent decrease in CO_2_ with CH_4_. Currently, the cause for this phenomenon remains unknown.

### 4.4. Bromoform Residue Deposition in Tissue

Although data on bromoform concentration in tissues and excretions are limited, Vucko et al. [16] reported that bromoform concentrations in *A. taxiformis* were affected by processing method. Methods in that study included a combination of rinsing in fresh vs. salt water, dipping vs. submerging for 3 or 6 min, freezing vs. not freezing, and kiln drying vs. freeze drying vs. dehydrating. However, regardless of rinsing method or time exposed to water, kelp biomass that was frozen and then freeze-dried consistently yielded higher bromoform concentrations when compared to other drying approaches [16], suggesting a potential effect on bromoform volatilization. Additionally, bromoform was detected in rinse water, suggesting that rinsing may also affect concentrations of this secondary metabolite in post-processed kelp [16]. 

As with other trihalomethane compounds, bromoform is readily absorbed through skin contact, breathing, and ingestion, although ingestion is the primary route of entry [24]. The CDC has identified bromoform as a human carcinogen. Thus, it is important that residues are not retained in animal tissues. In animal studies, repeated oral exposure to 600 mg·kg^−1^·d^−1^ of bromoform resulted in 100% mortality in rats, whereas no deaths were reported when exposure was decreased to 400 mg·kg^−1^·d^−1^ [25]. When administered above 100 mg·kg^−1^·d^−1^, carcinogenic risks of receiving bromoform orally five days per week for two years have also been reported in mice [25]. Due to their fat-soluble and hydrophobic nature, many halogenated organic compounds, such as bromoform, accumulate in the lipid components of human blood and milk [26]. Bromoform was detected in liver, intestine, and kidney samples from rodents provided water dosed with this trihalomethane compound [27]. Bromoform is predominantly processed by the liver although no significant effects on liver function, or biochemical or histological changes, were observed in mice when bromoform was administered interperitoneally at 25–300 µL/kg as an oil suspension (corn oil 1 mL/kg; [28]). Intraperitoneal injection of 1 mmol/kg bromoform, using corn oil as a vehicle, increased carbon monoxide concentrations in the blood of male rats through a cytochrome p450 mechanism [29,30,31]. Carbon dioxide is also commonly referenced to as a metabolite of bromoform [30], as several halothanes were observed to increase exhaled CO_2_ concentrations in rodents [32]. Following the metabolism of bromoform via cytochrome p450, both CO and CO_2_ are expired through normal respiratory functions.

Oral bromoform administration during this study did not contribute to detectable accumulations of bromoform within the liver (limit of detection = 8.5 µg/kg). This suggests that, following hepatic processing, bromoform may be retained within hepatic tissue as a metabolite or metabolized further elsewhere. Although bromoform was not detected in liver, kidney, muscle, and adipose tissue collected in this study following 30 d of supplementation, it is important to consider potential metabolites of bromoform, such as bromide, as targets for investigation. Increasing dosages of bromoform up to 1600 mg were administered to non-pregnant Holstein–Friesian heifers both orally and intravenously, with a rapid decrease in blood concentrations of this trihalomethane compound noted for all doses, regardless of administration method, during the first 24 h [33]. However, the authors of that study noted that clearance of bromoform was slower when doses were above 400 mg, indicating a threshold for metabolism, elimination, or absorption capacity. The detectable blood concentrations in the Bhusal et al. [33] study compared with the absence of detectable bromoform in tissues of cattle from the current study may relate to the administration method of the compound, which could have differential effects on the rate at which bromoform is introduced into circulation. In our study, the mixing of this compound in the TMR, rather than as a drench, as in Bhusal et al. [33], may have allowed for a slower introduction of bromoform into circulation, leading to more rapid clearance rates consistent with lower dosages administered via drench and intravenous methods in the Bhusal et al. study [33].

Bromide has been identified in tissues following exposure to bromoform, but few studies have evaluated its retention following bromoform supplementation. These binary compounds are primarily excreted through the renal pathway, with reabsorption of bromide commonly observed when chloride intake is low [34,35]. In addition to bromide, other major metabolites of bromoform include dibromochloromethane, bromochloroacetic acid, and dibromoacetic acid [16].

Halogenated organic compounds were identified in maternal human breastmilk of individuals exposed to bromoform by Pellizzari et al. [36]. Similarly, Muizelaar et al. [37] reported this compound as potentially problematic when *A. taxiformis* was fed to lactating cattle, as bromoform was detected in milk collected from dairy cows supplemented with varying levels of bromoform. That study also reported histological changes to the rumen wall of two of the cows harvested during the study, including a reduction in ruminal papillae, increased invasion of inflammatory cells, and formation of ulcers. Bromoform was detected in the milk of low- and medium-supplemented animals after 1 d of treatment, and in urine for all treatments (limits of detection 6–267 µg/L; [37]). The absence of detectable residues in feces throughout that study, as well as the presence of bromoform in milk and urine samples, suggests that bromoform does not accumulate in tissues, but may instead be excreted via urine and milk [37]. However, Roque et al. [15] reported absence of bromoform residues in milk from lactating dairy cows consuming 6.6 ppm and 13.2 ppm bromoform through *A. armata* dietary supplementation, suggesting bovine physiology may allow for preferential excretion of this methane-mitigating compound as waste below a certain threshold before it accumulates in consumable animal products (i.e., milk). Following 30 d of PKBP supplementation in Experiment 3, no significant bromoform residues were detected in any of the tissues (liver, kidney, muscle or adipose) across treatments, and limits of detection ranged from 3.7 to 9.1 µg/kg, lower than those described in previous studies. The findings of the present research suggest that bromoform is not retained in the tissue following product supplementation. However, future research should focus on the potential accumulation of bromoform metabolites resulting from supplementation of this halogenated methane analogue.

## 5. Conclusions

Dietary PKBP supplementation decreased CH_4_ production and yield without affecting DMI or whole animal oxidative metabolism, suggesting dietary inclusion of this proprietary bromoform product may be a viable option for the mitigation of CH_4_ emissions in cattle. Supplementing bromoform at 20 ppm of DMI reduced CH_4_ production and maintained lower CH_4_ production throughout the 9 d period following cessation of supplementation. Although changes were observed in CO_2_ production and O_2_ consumption at 20 ppm bromoform supplementation, respiratory quotient, DMI, ADG, and gain:feed remained unaffected. Thus, at this time we do not believe that the observed changes are detrimental to the animals.

Although we did not detect effects of bromoform on DMI, ADG, or feed conversion efficiency, these measures were ancillary to our primary objectives of evaluating effects on CH_4_ and tissue residues. There is weak evidence from this study that DMI (and therefore ADG and G:F) could be affected by bromoform, and these are legitimate targets for future studies. No bromoform in kidney, liver, longissimus dorsi muscle tissue, or adipose tissue was detected across treatments, suggesting that the supplementation of bromoform of up to 20 ppm in daily rations for 30 d to growing cattle does not contribute to significant accumulations of this trihalomethane compound within tissues. Future research should focus on whether residual CH_4_ suppression exceeds that observed in the current study, and on the accumulation of bromoform metabolites, including bromide, in tissues.

## Figures and Tables

**Table 1:** Basal ration, by experiment, provided to steers during investigations of supplementing a dietary proprietary kelp blend product (PKBP) containing metabolites, including bromoform ^a^.

	Experiments		
Feedstuff	1 ^b^	2 ^b^	3 ^c^
Corn Silage	62.9	52.6	52.6
Cracked Corn	0	21.1	21.1
Distillers Dried Grains	28.5	15.8	15.8
Soybean Meal	6.1	0	0
Calcium Carbonate	1.7	0	0
Vitamin and Mineral Supplement ^d^	0.7	10.5	10.5

^a^ Bromoform inclusion rates for each study are listed, by experiment, in the materials and methods. ^b^ Steers were restriction fed at 1.5 × NE_m_ in Experiments 1 and 2. ^c^ Steers were provided feed ad libitum in Experiment 3. ^d^ Mineral supplement included at a level to provide adequate levels of Na, Cl, S, Se, I, Zn, Mn, Co, and Cu as well as vitamins A, D, E for meeting dietary recommendations set by NASEM [11] for growing cattle.

**Table 2:** Effects of dietary treatment with a proprietary kelp blend product (PKBP) containing metabolites, including bromoform at 0 and 52.5 ppm, on dry matter intake of steers during Experiment 1 adaptation and respiratory gas collection periods ^a^.

		Treatment			*p*-Value	
Item	Period	Control	Bromoform	SE	Block	Treatment
Dry matter intake, kg	Diet adaptation ^b^	3.39	3.34	0.027	<0.01	0.20
	Gas collection ^c^	3.35	3.21	0.045	<0.01	0.04
Dry matter intake, kg/kg BW	Diet adaptation ^b^	0.021	0.021	0.0002	0.1	0.17
	Gas collection ^c^	0.021	0.020	0.0021	0.51	0.11

^a^ Data are presented as least square means ± the standard error of the mean; *n* = 6 and *n* = 5 for the control and PKBP supplemented treatment, respectively. ^b^ Diet adaptation period was 11 d. ^c^ Gas collection period was 3 d.

**Table 3:** Effects of dietary treatment with PKBP on Experiment 1 respiratory gas production (L/d) and oxygen consumption (L/d) in steers ^a^.

	Treatment			*p*-Value	
Item	Control	Bromoform	SE	Block	Treatment
CH_4_, L/d	88.7	<2.2 ^b^	7.5	0.33	<0.001
O_2_, L/d	1473	1606	65.9	0.41	0.20
CO_2_, L/d	1500	1595	60.8	0.28	0.31

^a^ Data are presented as least square means ± the standard error of the mean; *n* = 6 and *n* = 5 for the control and PKBP treatment, respectively. ^b^ All measures were below detection limits. Estimated maximal production based on a sensitivity of 2 ppm.

**Table 4:** Experiment 2 dietary PKBP inclusion effects across days on post-supplementation CH_4_ production ^a,b^.

				Treatment		Day (Treatment)		
Item	Treatment	Mean	SEM	Linear	Quad.	Linear	Quad.	Cubic
CH_4_, L/day	Control	206	6.6	<0.01	0.04	0.63	0.42	0.20
	9.5 ppm	184				0.19	0.44	0.11
	20 ppm	115				<0.01	<0.01	0.02
CH_4_ yield, g/kg DMI	Control	22.88	0.557	<0.01	0.08	0.47	0.46	0.13
	9.5 ppm	19.80				0.11	0.34	0.09
	20 ppm	13.86				<0.01	<0.01	0.01
CH_4_, g/kg BW	Control	0.41	0.0127	<0.01	0.01	0.48	0.31	0.16
	9.5 ppm	0.37				0.07	0.33	0.07
	20 ppm	0.26				<0.01	<0.01	<0.01

^a^ Data are presented as least squares means ± the SEM; *n* = 2 for control, *n* = 2 for 9.5 ppm, *n* = 2 for 20 ppm bromoform inclusion in daily ration. ^b^ Interaction of treatment and day was significant (*p* < 0.01) for all three measures of CH_4_.

**Table 5:** Experiment 2 dietary PKBP effects across treatments by day on post-supplementation CH_4_ production ^a,b^.

		Treatments				Treatment (Day)	
	Day	Control	9.5 ppm	20 ppm	SEM	Linear	Quad.
CH_4_, L/d	1	201.5	159.7	4.4	13.05	<0.01	<0.01
	2	210.5	171.5	22.2	13.05	<0.01	<0.01
	3	202.4	183.8	123.4	13.05	<0.01	0.21
	4	204.2	190.3	144.8	13.05	<0.01	0.34
	5	211.2	202.6	150.0	13.05	<0.01	0.19
	6	195.7	178.6	138.5	13.05	<0.01	0.48
	7	201.0	186.5	154.2	13.05	0.02	0.58
	8	201.1	177.2	139.3	13.05	<0.01	0.67
	9	228.3	205.2	157.5	13.05	<0.01	0.45
CH_4_ Yield, g/kg DMI	1	21.9	16.8	0.5	1.32	<0.01	<0.01
	2	23.5	18.6	2.7	1.32	<0.01	<0.01
	3	22.5	19.9	14.9	1.32	<0.01	0.48
	4	22.8	20.5	17.5	1.32	<0.01	0.79
	5	23.5	21.8	18.1	1.32	<0.01	0.53
	6	21.8	19.3	16.7	1.32	0.01	0.98
	7	22.3	20.1	18.6	1.32	0.06	0.84
	8	22.3	19.1	16.8	1.32	<0.01	0.78
	9	25.4	22.1	19.0	1.32	<0.01	0.96
CH_4_, g/kg BW	1	0.6	0.4	0.0	0.03	<0.01	<0.01
	2	0.6	0.5	0.1	0.03	<0.01	<0.01
	3	0.6	0.5	0.4	0.03	<0.01	0.33
	4	0.6	0.5	0.5	0.03	0.02	0.58
	5	0.6	0.6	0.5	0.03	0.02	0.34
	6	0.5	0.5	0.4	0.03	0.02	0.80
	7	0.6	0.5	0.5	0.03	0.12	0.99
	8	0.6	0.5	0.4	0.03	0.01	0.93
	9	0.6	0.6	0.5	0.03	<0.01	0.80

^a^ Data are presented as least squares means ± the SEM; *n* = 2 for control, *n* = 2 for 9.5 ppm, *n* = 2 for 20 ppm bromoform inclusion in daily ration. ^b^ Interaction of treatment and day was significant (*p* < 0.01) for all three measures of CH_4_.

**Table 6:** Effects of dietary PKBP supplementation across days during Experiment 2 on CO_2_ production, O_2_ consumption, and respiratory quotient ^a,b^.

				*p*-Values					
					Contrasts				
					Treatment		Day (Treatment)		
Item	Treatment	Mean	SEM	Day	Linear	Quad.	Linear	Quad	Cubic
CO_2_, L/day	Control	3104	78.6	0.26	<0.01	0.02	0.86	0.27	0.16
	9.5 ppm	3159					0.26	0.23	0.65
	20 ppm	2543					0.47	0.98	0.63
CO_2_, g/kg BW	Control	17.01	0.468	0.08	<0.01	<0.01	0.72	0.13	0.12
	9.5 ppm	17.51					0.03	0.10	0.59
	20 ppm	15.93					0.11	0.95	0.57
O_2,_ L/day	Control	3032	84.1	0.35	<0.01	0.02	0.79	0.05	0.27
	9.5 ppm	3129					0.15	0.10	0.58
	20 ppm	2504					0.85	0.36	0.45
O_2,_ g/kg BW	Control	12.08	0.397	0.27	<0.01	<0.01	0.66	0.03	0.27
	9.5 ppm	12.61					0.03	0.06	0.58
	20 ppm	11.40					0.75	0.21	0.39
RQ	Control	1.02	0.008	0.37	0.2	0.08	0.87	0.12	0.30
	9.5 ppm	1.01					0.62	0.64	0.06
	20 ppm	1.02					<0.01	0.02	0.62

^a^ Data are presented as least squares means ± the SEM; *n* = 2 for control, *n* = 2 for 9.5 ppm, *n* = 2 for 20 ppm bromoform inclusion in daily ration. ^b^ Interaction of treatment and day was significant (*p* ≤ 0.02) for all measures of CO_2_ and O_2_, but not RQ (*p* = 0.08).

**Table 7:** Effects of dietary PKBP supplementation during Experiment 3 on dry matter intake and growth performance measures of Angus steers ^a^.

	Treatments				*p*-Value		
		Bromoform Inclusion				Contrasts	
Item	Control	9.5 ppm	20 ppm	SEM	Trt	Linear	Quadratic
Initial BW, kg	445	395	420	17.5	0.17	0.37	0.13
DMI, kg	11.13	9.46	9.34	0.310	<0.01	<0.01	0.08
DMI, %BW	2.38	2.28	2.11	0.139	0.47	0.23	0.82
ADG, kg/d	1.67	1.41	1.53	0.18	0.55	0.60	0.41
Growth efficiency, g/kg	150.5	148.2	163.5	16.3	0.82	0.60	0.67

^a^ Data are presented as least squares means ± the SEM; *n* = 2 for control, *n* = 2 for 9.5 ppm, *n* = 2 for 20 ppm bromoform inclusion in daily ration.

**Table 8:** Bromoform residues in organ, adipose and muscle tissue samples following 30 d supplementation period of Experiment 3.

	Control	9.5 ppm Bromoform	20 ppm Bromoform	
Item	Bromoform, µg/kg ^a^			LOD, µg/kg ^b^
Liver	<LOD	<LOD	<LOD	8.5
Kidney	<LOD	<LOD	<LOD	9.1
Adipose	<LOD	<LOD	<LOD	3.7
Muscle	<LOD	<LOD	<LOD	4.4

^a^ *n* = 4 for control, *n* = 3 for 9.5 ppm, *n* = 2 for 20 ppm bromoform inclusion in daily ration. ^b^ LOD = limit of detection.

## Data Availability

Dataset available on request from the authors.
